# Beyond social skills: emotion regulation as a potential mechanism linking physical exercise to social anxiety in left-behind adolescents

**DOI:** 10.3389/fpsyg.2026.1753268

**Published:** 2026-10-29

**Authors:** Li Wu, Yiqiong Huang, Ran Bao, Lin Jiangfeng, Ran Duan

**Affiliations:** 1 Faculty of Teacher Education, Zhaoqing University, Zhaoqing, Guangdong, China; 2 School of Education, City University of Macau, Taipa, Macao SAR, China; 3 School of Education, Zhaoqing University, Guangdong, China; 4 Faculty of Education, University of Macau, Taipa, Macao SAR, China

**Keywords:** emotion regulation, gender differences, left-behind adolescents, mediation, physical exercise, social anxiety

## Abstract

**Introduction:**

This cross-sectional, exploratory study investigated the association between physical exercise (PE) and social anxiety (SA) among 862 left-behind adolescents in China. It examined emotion regulation abilities (ERA) and peer relationships (PR) as potential mediators and gender as a moderator in this association.

**Methods:**

A cross-sectional exploratory design was adopted. Participants were 862 Chinese left-behind adolescents. Path analysis was used to test the hypothesized sequential mediation model and gender moderation effect.

**Results:**

Path analysis revealed that PE was negatively associated with SA. Each separate path of the hypothesized sequential chain (PE → ERA, ERA → PR, PR → SA) was statistically significant, while the overall sequential mediation effect (PE → ERA → PR → SA) was non-significant (95% CI included zero). This indicated that the significant PE–ERA association had a small effect size and could not propagate fully through the chain to SA. The direct path from PE to PR was also non-significant. The data favoured independent mediation pathways rather than a sequential mediation process. Gender moderated the pathway: the positive PE–ERA association was significant for females but non-significant for males.

**Discussion:**

The findings are hypothesis-generating rather than confirmatory and require replication via longitudinal research. For vulnerable adolescents, PE may help build emotional competence, which contributes to improved peer bonds. Interventions could integrate emotion-regulation skill training into physical activity programmes, particularly targeting female adolescents. Due to the cross-sectional nature of the study, causal conclusions cannot be drawn.

## 1 Introduction

The global phenomenon of labor migration has resulted in a significant population of “left-behind children”—minors living without one or both parents for extended periods. This issue is particularly pronounced in developing regions, impacting millions of children worldwide (UNICEF, 2018). For instance, recent data show that 31% of children in Moldova and 27% of youth in the Philippines are affected by parental migration (Cebotari et al., 2018; Madianou and Miller, 2011). China represents a particularly stark example, with an estimated 66 million left-behind children in 2020 (Lyu et al., 2024). The prolonged absence of parental care during critical developmental stages places these children at a heightened risk for emotional and psychological difficulties.

Among the various mental health challenges, social anxiety (SA)—characterized by intense fear or discomfort in social situations—is especially prevalent in this demographic. Meta-analytic evidence reveals that the incidence of SA among China’s left-behind adolescents is 36.1%, significantly higher than the 20.2% observed in their non-left-behind peers (Li, 2017, 2019). Adolescence is a period of profound physical and psychosocial transition, where accelerated physical growth often contrasts with still-developing psychological maturity (He, 2020). For left-behind adolescents, this developmental dissonance is compounded by a lack of parental guidance and emotional support, making them exceptionally vulnerable to developing SA (Zeng et al., 2014).

Physical exercise (PE) has emerged as an accessible and effective non-clinical intervention for improving mental wellbeing. A body of evidence, including systematic reviews of randomized controlled trials, suggests that regular PE may alleviate anxiety symptoms across various populations (Jayakody et al., 2014; Thompson et al., 2019). Preliminary studies on left-behind adolescents align with these findings, suggesting that PE may act as a protective factor against SA (Li et al., 2020). Based on this established negative association, we propose the first hypothesis:

*Hypothesis 1 (H1)*: Physical exercise may be negatively associated with social anxiety in left-behind adolescents.

While the link between PE and reduced SA is well-documented, the underlying mechanisms that explain how this process unfolds for left-behind adolescents are not well understood. This study proposes two critical psychosocial mediators. First, we consider emotion regulation abilities (ERA), which involve managing emotional responses. Left-behind adolescents often exhibit poorer ERA due to stress and lack of parental modeling (Huang and Zhang, 2015), which in turn contributes to anxiety. Conversely, PE is known to enhance ERA, Based on Gross’s process model of emotion regulation, it has been theorized that physical exercise may optimize the cognitive appraisal and expressive suppression processes of emotion regulation, potentially improving the ability to manage negative emotions (Gross, 2002). Possibly by modulating neurobiological systems and providing a constructive outlet for negative emotions (Giles et al., 2017). Therefore, it is plausible that PE reduces SA by first improving an adolescent’s ability to regulate their emotions. This leads to our second hypothesis:

*Hypothesis 2 (H2)*: Emotion regulation abilities may mediate the association between physical exercise and social anxiety.

Second, we examine the role of peer relationships (PR). For adolescents, and especially for those lacking daily parental interaction, peers become a primary source of social support and identity validation (Harris, 1995). Poor PR is a significant risk factor for SA (Pickering et al., 2020). Participation in PE, particularly team-based activities, creates structured opportunities for social interaction, facilitating cooperation and strengthening peer bonds (Han et al., 2019). This enhanced social connection can directly mitigate the feelings of isolation and fear inherent in SA. Thus, we hypothesize:

*Hypothesis 3 (H3)*: Peer relationships may mediate the association between physical exercise and social anxiety.

From the perspective of social information processing theory, individuals with high emotion regulation ability can accurately perceive and interpret social cues in peer interactions, reduce negative emotional responses to interpersonal conflicts, and thus promote the establishment and maintenance of high-quality peer relationships (Crick and Dodge, 1994). Building upon these individual pathways, this study posits a more nuanced, sequential mechanism. We theorize that ERA is proposed as a potential foundational skill for successful social functioning. An adolescent who can effectively regulate their emotions is better equipped to navigate interpersonal challenges, communicate effectively, and consequently build and maintain higher-quality peer relationships. This suggests that the impact of PE on social outcomes is not direct, but is first enabled by internal, emotional changes. This leads to our primary and most novel hypothesis, a chain mediation model:

*Hypothesis 4 (H4)*: Emotion regulation abilities and peer relationships will sequentially mediate the association between physical exercise and social anxiety.

This hypothesis rests on the assumption that the PE → ERA association is sufficiently strong to propagate through ERA→PR and PR → SA. Should this hypothesis be rejected, the pattern of individual path significance will inform whether the failure lies in the sequential logic itself or in the magnitude of the initial PE → ERA link.

In summary, the present study aims to test this comprehensive chain mediation model (see Figure 1). By elucidating these specific pathways, we seek to move beyond simply confirming the association between PE and reduced SA, to explaining how it works. This may enhance the theoretical understanding of SA among vulnerable adolescents but also provide a more targeted and evidence-based foundation for designing effective school-based interventions.

**Figure 1:**
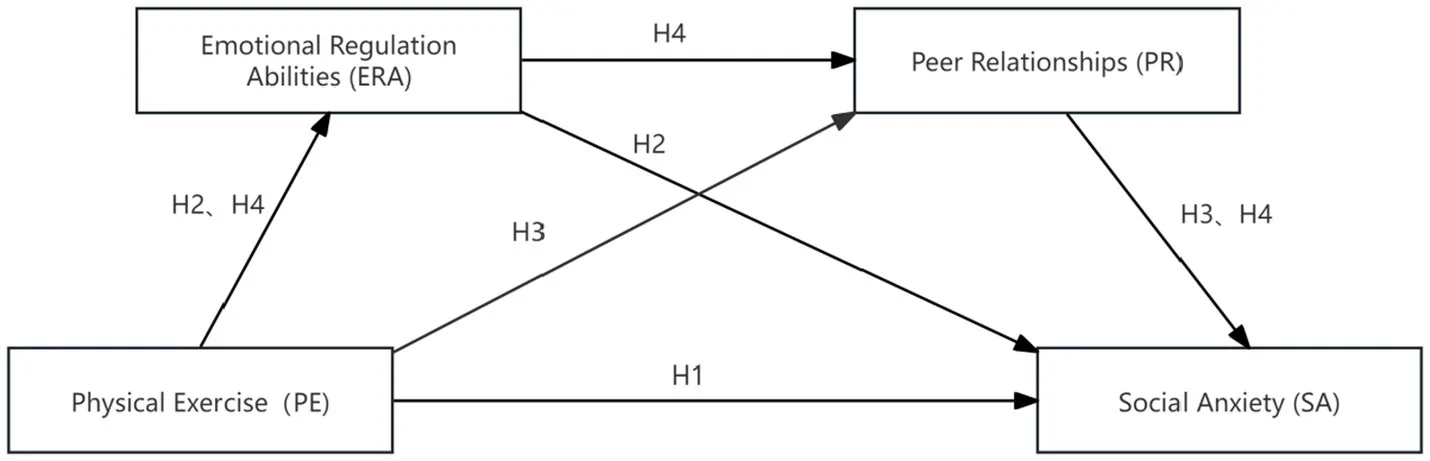
Hypothesis model.

## 2 Methods

### 2.1 Participants and procedure

This study employed a convenience sampling method to recruit participants from several middle schools in Zhaoqing and Yunfu, two developing regions in Guangdong Province, China. The selected schools are known to have a substantial enrollment of left-behind adolescents. This convenience sampling strategy may limit the generalizability of the study results to the broader population of left-behind adolescents in China.

Trained research assistants collected data in classrooms during scheduled periods. After receiving approval from school administrators, trained research assistants explained the study’s purpose and procedures to the students. The research assistants emphasized that participation was completely voluntary and that their responses would be kept anonymous and confidential. Written informed consent was obtained from all participants and their guardians prior to the survey administration.

A total of 2,000 paper-and-pencil questionnaires were distributed. Of these, 1,953 were returned (a 97.7% response rate). After removing incomplete or invalid responses, 1,652 questionnaires were retained. From this sample, we identified participants who met the criteria for being “left-behind,” operationally defined as an adolescent with at least one parent having migrated away from home for work for 6 months or longer. This screening yielded a final sample of 862 left-behind adolescents.

The final sample consisted of 364 males (42.2%) and 498 females (57.8%). The participants’ ages ranged from 12 to 16 years, with a mean age of 13.78 years (SD = 1.15). The grade distribution was as follows: 280 students in Grade 7 (32.5%), 312 in Grade 8 (36.2%), and 270 in Grade 9 (31.3%). This study was conducted in accordance with the Declaration of Helsinki, and was approved by the Ethics Committee of Zhaoqing University (No. 2024032).

### 2.2 Measures

All English scales were translated into Chinese following the standardized back-translation procedure (Brislin, 1970): first, two bilingual researchers independently translated the original scales into Chinese; then, another two bilingual experts blind to the original scales back-translated the Chinese version into English; finally, the research team and two psychology experts compared the original and back-translated versions, revised inconsistent items, and formed the final Chinese version. A pilot test was conducted with 100 left-behind adolescents, and the scale items were adjusted according to the pilot test results to ensure the validity and readability of the scales. All variables were measured using established scales translated into Chinese. Responses were recorded on a 5-point Likert scale.

*Physical Exercise (PE)*. PE was assessed using the Physical Activity Rating Scale (PARS-3). This 3-item scale measures the intensity, duration, and frequency of exercise over the past month. A sample item is, “How often did you participate in physical exercise each week?”. Higher scores indicate greater engagement in physical exercise. The scale showed acceptable internal consistency in the current study (*α* = 0.850).

*Social Anxiety (SA)*. SA was measured with the 6-item Interaction Anxiousness Scale, a subscale of the Self-Consciousness Scale revised for Chinese samples. A sample item is, “I feel nervous when I have to speak to someone in a position of authority”. Higher total scores reflect a higher level of social anxiety. The scale showed strong internal consistency (*α* = 0.871).

*Emotion Regulation Abilities (ERA)*. We used the “effective application of emotional regulation strategies” subscale from the Adolescent Emotional Regulation Questionnaire to assess ERA. This 5-item subscale measures an individual’s perceived effectiveness in using strategies to manage emotions. A sample item is, “When I am in a bad mood, I can quickly find a way to make myself feel better.” Higher scores indicate better emotion regulation abilities. The Cronbach’s alpha for this scale was 0.692, which is below the conventional threshold of 0.70. McDonald’s omega was calculated as a supplementary reliability estimate and yielded a value of 0.74, suggesting acceptable internal consistency (Dunn et al., 2014). CFA showed acceptable model fit (*χ^2^*/df = 3.18, CFI = 0.89, TLI = 0.87, RMSEA = 0.055), supporting the scale’s construct validity in this sample. The marginal internal consistency of this measure is acknowledged as a study limitation and is addressed in the limitations section.

*Peer Relationships (PR)*. PR were assessed using the “affective interactions” subscale of the Peer Relationship Scale for Children and Adolescents. This 5-item scale measures the quality of emotional connection and support among peers. A sample item is, “I feel happy when I am with my friends.” Higher scores indicate better peer relationships. The Cronbach’s alpha for this scale was 0.693, and McDonald’s omega was 0.72. CFA showed acceptable model fit (*χ^2^*/df = 3.09, CFI = 0.90, TLI = 0.88, RMSEA = 0.053). The marginal reliability is acknowledged as a limitation.

A limitation of this study is that the internal consistency of the ERA (*α* = 0.692) and PR (*α* = 0.693) scales fell slightly below the conventional threshold of 0.70. While such values are marginally acceptable in exploratory research (Hair et al., 2010), they may have attenuated the observed effect sizes and reduced the statistical power of the mediation analyses. Therefore, the findings involving ERA and PR should be interpreted with caution, and future studies should employ more robust psychometric instruments to replicate these results (Hair et al., 2010).

### 2.3 Data analysis

Data were analyzed using SPSS 26.0. First, we conducted preliminary analyses, including descriptive statistics, Pearson correlation analyses for all study variables, and independent samples t-tests and ANOVAs to examine group differences. Harman’s single-factor test was performed to check for common method bias. In addition to Harman’s single-factor test, we also adopted the unmeasured latent method factor approach to further test common method bias. The results showed that the model fit did not improve significantly after adding the latent method factor (ΔCFI <0.01), indicating that common method bias was not a serious issue in this study.

The primary analysis involved testing the hypothesized chain mediation model using the PROCESS macro for SPSS (Model 6). This model simultaneously tests the direct effect of PE on SA and the three proposed indirect pathways:

Indirect effect via ERA (PE → ERA→SA).

Indirect effect via PR (PE → PR → SA).

The sequential indirect effect via ERA and then PR (PE → ERA→PR → SA).

The significance of the indirect effects was determined by constructing 95% bias-corrected bootstrap confidence intervals based on 5,000 resamples. An indirect effect is considered statistically significant if its 95% confidence interval does not contain zero. To ensure methodological rigor, all analyses controlled for the potential confounding effects of age, gender, and grade. All variables in the mediation model were standardized prior to analysis. Standardized coefficients are reported in the main text and figures, and unstandardized coefficients with standard errorsare reported in the tables for reference.

## 3 Results

### 3.1 Preliminary analyses

A Harman’s single-factor test was conducted to assess common method bias. The results yielded four factors with eigenvalues greater than 1, and the first unrotated factor accounted for 34.2% of the variance. As this is below the 40% threshold, common method bias was not considered a significant issue in this study. The unmeasured latent method factor approach further confirmed that the common method bias was within an acceptable range (the total variance explained by the method factor was 8.7%), which supported the reliability of the study results.

Descriptive statistics and Pearson correlation coefficients for all study variables are presented in Table 1. As hypothesized, PE was positively and significantly correlated with ERA (*r* = 0.102, *p* < 0.01) and PR (*r* = 0.096, *p* < 0.01), and negatively correlated with SA (*r* = −0.140, *p* < 0.01). The magnitudes of these correlations were small (*r* ≈ 0.10). Given the large sample size (*N* = 862), statistical significance should be interpreted with caution; the practical significance of these associations is limited. The interpretation of these small effect sizes is addressed in the limitations section. ERA and PR were also strongly and negatively correlated with SA.

**Table 1:** Descriptive statistics and Pearson correlations.

	Variable	Mean	SD	1	2	3	4
1	Physical Exercise (PE)	2.78	0.71				
2	Social Anxiety (SA)	2.89	0.94	−0.140^**^			
3	Emotion Regulation (ERA)	3.30	0.86	0.102^**^	−0.583^**^		
4	Peer Relationships (PR)	3.10	0.81	0.096^**^	−0.666^**^	0.503^**^	

^**^p < 0.01.

Table 2 displays the means and standard deviations for all variables, disaggregated by gender. Independent samples t-tests revealed significant gender differences. Compared to females, males reported significantly higher levels of PE, ERA, and PR, and significantly lower levels of SA (all *p* < 0.001).

**Table 2:** Gender differences.

Variable	Male	Female	*t*	*p*
	*M* (SD)	*M* (SD)		
PE	2.98 (0.71)	2.63 (0.67)	8.03	<0.001
SA	2.63 (0.94)	3.07 (0.90)	−7.59	<0.001
ERA	3.41 (0.87)	3.21 (0.84)	3.79	<0.001
PR	3.24 (0.82)	3.00 (0.78)	4.88	<0.001

df = 860 for all comparisons.

^**^p < 0.01.

### 3.2 Chain mediation analysis

Based on the hypothetical model (Figure 1), we tested the hypothesized chain mediation model using PROCESS Model 6, with age, gender, and grade entered as covariates. The results of the path analysis are detailed in Figure 2 and show partial support for our hypotheses.

**Figure 2:**
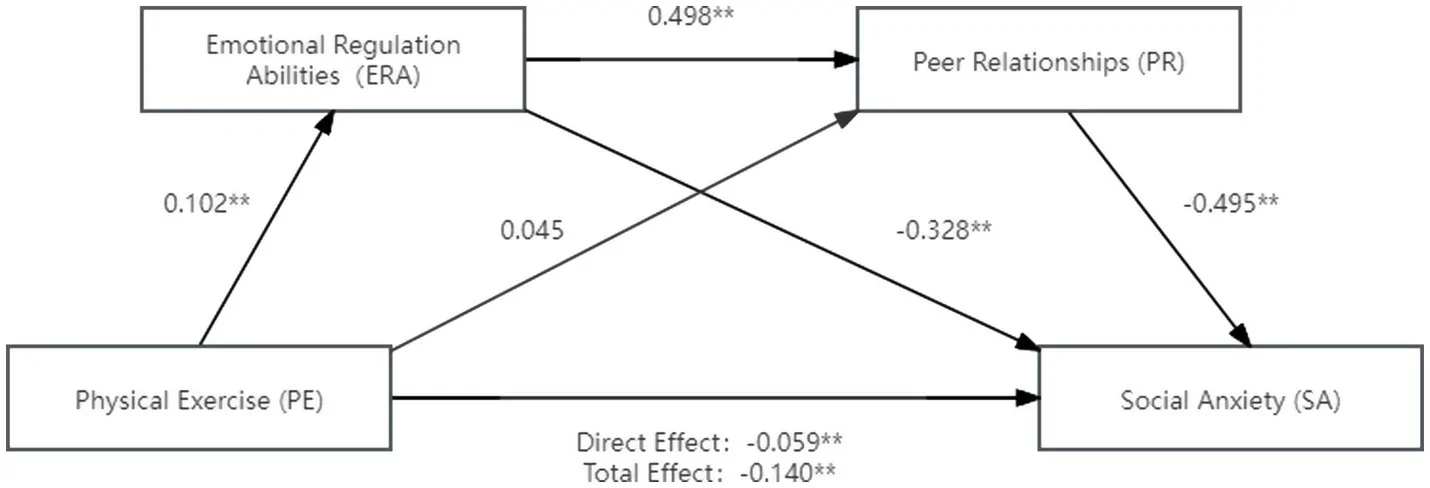
Path diagram of the chain mediation model. PE = Physical Exercise; PR = Peer Relationships; ERA = Emotional Regulation Abilities; SA = Social Anxiety. Solid arrows indicate significant paths. Numbers on paths are standardized regression coefficients (*β*). The non-significant direct path from PE to PR (*β* = 0.045) is displayed. *p < 0.05; **p < 0.01; ***p < 0.001. Direct effect (PE → SA) = −0.059; Total effect = −0.140. The gender interaction term predicting ERA was significant (*β* = 0.087, *p* = 0.021).

The total effect of PE on SA was significant (*β* = −0.140, *p* < 0.001). The direct effect of PE on SA remained significant after accounting for the mediators (*β* = −0.059, *p* = 0.016). As predicted, PE had a significant positive effect on ERA (*β* = 0.102, *p* = 0.004), and ERA had a significant positive effect on PR (*β* = 0.498, *p* < 0.001). Both ERA (*β* = −0.328, *p* < 0.001) and PR (*β* = −0.495, *p* < 0.001) were significant negative predictors of SA. However, contrary to our initial expectation, the direct path from PE to PR was not significant (*β* = 0.045, *p* = 0.141). This pattern suggests that ERA may fully mediate the relationship between PE and PR.

The specific indirect effects were examined using bootstrapping (see Table 3). The indirect effect of PE on SA through ERA was significant (Effect = −0.034, 95% CI [−0.059, −0.010]), supporting H2. The indirect effect through PR was also significant (Effect = −0.022, 95% CI [−0.044, −0.008]), supporting H3.

**Table 3:** Bootstrap results for indirect effects of PE on SA.

Effect type	Path	Standard effect	SE	95%CI		Result
				LLCI	ULLCI	
Total effect (H1)	PE → SA (total)	−0.140	0.035	−0.210	−0.071	Supported
Indirect via ERA (H2)	PE → ERA → SA	−0.034	0.012	−0.059	−0.010	Supported
Indirect via PR (H3)	PE → PR → SA	−0.022	0.009	−0.044	−0.008	Supported
Sequential (H4)	PE → ERA → PR → SA	−0.025	0.020	−0.051	0.027	Not supported

SE = Standard Error; LLCI = Lower 95% Confidence Interval; ULLCI = Upper 95% Confidence Interval.

Notably, the sequential indirect effect from PE to ERA to PR to SA was not statistically significant (Effect = −0.025, 95% CI [−0.051, 0.027]). Therefore, H4 was not supported. This finding represents a meaningful empirical contribution, as it challenges the assumption that PE’s association with SA operates through a sequential social-cognitive cascade. Instead, the data pattern suggests that ERA and PR may function as independent mediators, though this parallel pattern emerged from post-hoc observation rather than *a priori* hypothesis testing.

### 3.3 Analysis of gender differences

To explore potential gender differences in the model’s pathways, we examined the moderating role of gender. The results indicated a significant moderation on the path from PE to ERA. Specifically, the positive effect of PE on ERA was significant for females (*β* = 0.110, *p* = 0.023) but was not significant for males (*β* = 0.039, *p* = 0.474). This pattern suggests that physical exercise may be more strongly associated with improved emotion regulation for improving emotion regulation among left-behind female adolescents than their male counterparts. To further test the gender moderation effect, we conducted a multi-group SEM analysis and tested the interaction term (PE × Gender) predicting ERA. The results showed that the interaction term was significant (*β* = 0.087, *p* = 0.021), confirming that the effect of PE on ERA was significantly different between males and females (see Figure 3 for the interaction plot).

**Figure 3:**
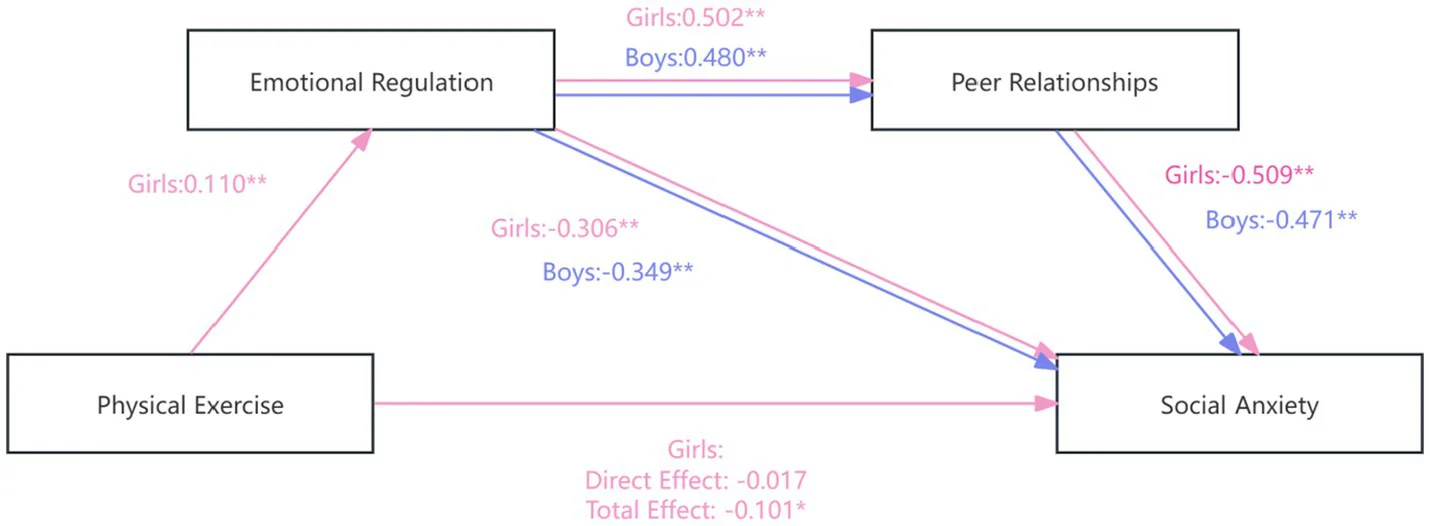
Differences in gender. PE = Physical Exercise; PR = Peer Relationships; ERA = Emotional Regulation Abilities; SA = Social Anxiety. Blue = male; pink = female. Coefficients are standardized *β*. ^*^p < 0.05; ^**^p < 0.01; ^***^p^*^ < 0.001. Significant paths: PE → PR (0.480^**^ male/0.110^**^ female); PR → ERA (0.480^**^ male/0.502^**^ female); PR → SA (−0.349^**^ male/–0.306^**^ female). Total effect PE → SA: −0.101^*^ (females only).

The significant gender moderation (PE → ERA only for females) may be understood through multiple complementary lenses. First, from a socialization perspective, female adolescents in Chinese cultural contexts are often socialized to be more emotionally expressive and interpersonally oriented (Li et al., 2017). Consequently, they may be more attuned to internal affective states and more likely to use exercise as an emotion regulation strategy. Second, from a stress-response perspective, research on gender differences in stress reactivity suggests that females tend to exhibit a “tend-and-befriend” pattern, which emphasizes social affiliation and emotional coping, whereas males more often display a “fight-or-flight” response (Rose and Rudolph, 2006). This difference may predispose female adolescents to intentionally engage in physical exercise as a means of managing emotional distress, thereby deriving greater regulatory benefits. Third, the type and context of exercise participation may differ systematically by gender. Research indicates that adolescent males are more likely to participate in competitive, team-based sports that emphasize performance and social comparison, which may introduce social evaluative threat and potentially offset the emotional benefits of exercise. In contrast, adolescent females tend to prefer or benefit more from supportive, non-competitive, or self-paced activities (e.g., individual sports, yoga, or running) that may be more conducive to emotion regulation (Casey et al., 2017). While our study did not directly measure exercise type or participation motives, these differences offer a plausible explanation for why the PE → ERA pathway was significant only for females.

Importantly, the lack of a significant effect for males does not imply that exercise is ineffective for them. Rather, other mechanisms—such as self-esteem, body image, or social comparison—may be more relevant pathways linking PE to reduced social anxiety in male left-behind adolescents. Future studies should directly measure exercise type (e.g., competitive vs. cooperative, team vs. individual), participation motives (e.g., enjoyment, appearance, social affiliation), and gender-specific stress-coping strategies to clarify these differential pathways.

## 4 Discussion

Given the cross-sectional design, all findings should be interpreted as associative rather than causal. This exploratory, cross-sectional study investigated how PE is associated with SA among left-behind adolescents. Our findings reveal a nuanced pattern: while PE was negatively associated with SA (H1), and this association was independently mediated by both ERA (H2) and PR (H3), the hypothesized sequential mediation (PE → ERA→PR → SA; H4) was not supported. Critically, the non-significance of the chain mediation does not reflect a failure of the sequential logic, as each constituent path (PE → ERA, ERA→PR, and PR → SA) was individually significant. Rather, the PE → ERA association (*β* = 0.102) was too modest in magnitude to generate a significant product term through the chain. This pattern suggests that for left-behind adolescents, the bottleneck in the hypothesized cascade lies in the initial link between physical exercise and emotion regulation. Therefore, the current findings do not provide empirical evidence for a stepwise process linking emotion regulation to peer relationships and subsequently to social anxiety. Beyond the non-significant chain, the data also showed that ERA and PR functioned as independent mediators of the PE-SA association. However, this parallel pattern emerged from post-hoc observation following the rejection of H4, rather than from *a priori* hypothesis testing. It should therefore be interpreted as hypothesis-generating rather than confirmatory, and requires independent replication in future research. The primary contribution of this study, we suggest, lies not in establishing a new parallel model, but in identifying the PE → ERA link as the key bottleneck in the sequential pathway. Furthermore, the influence of PE on ERA was significantly stronger for females. These results challenge a simple linear model and instead suggest ERA as a primary mechanism in the process by which PE is associated with mental health benefits.

### 4.1 The primacy of emotion regulation (ERA) as a mediator

In terms of effect size, the indirect effect of PE on SA through ERA (Effect = −0.034) was larger than that through PR (Effect = −0.022), which suggested that ERA may play a more important mediating role in the relationship between PE and SA. The most consistent finding of this study is the mediating role of ERA. PE was significantly associated with higher ERA, which in turn predicted lower SA. This pattern is consistent with neurobiological theories suggesting that exercise may regulate arousal, manage stress responses, and enhance cognitive control over emotions by inducing neurostructural changes in brain regions like the cerebral cortex and hippocampus (Wang, 2021; Sun et al., 2023). Engaging in appropriate PE helps individuals release negative energy, mitigate negative emotions, and support psychological balance (Dunston, 2020; O’Brien et al., 2016). For left-behind adolescents, who often lack parental co-regulation, face heightened stress, and consequently exhibit poorer ERA (Huang and Zhang, 2015), PE could serve as a tool for self-regulation (Belcher et al., 2021; Zhang et al., 2024).

This pattern challenges a purely social-enhancement explanation of PE’s benefits. Instead, this pattern is consistent with a neurocognitive account: physical exercise may improve top-down emotion regulation by strengthening prefrontal control over limbic regions such as the amygdala (Smith and Merwin, 2021; Kandola et al., 2019), a pathway that is especially valuable for left-behind adolescents who lack consistent parental co-regulation.

Notably, this pathway was significantly stronger for females. Left-behind female adolescents in our sample reported lower levels of PE and ERA, yet they appeared to derive greater emotional regulatory benefits from exercise than their male counterparts. This could reflect gender differences in emotional processing or culturally patterned emotional socialization (Li et al., 2017), where females may be more attuned to internal affective states (Barrett et al., 2000), making them more responsive to interventions that target these processes. These findings are also consistent with research suggesting that exercise-sensitive neurobiological mechanisms in female emotion-regulation systems might explain these gains (Smith et al., 2020; Johnson & Taylor, 2021). This tentatively suggests that promoting PE, especially among girls, could be a particularly potent strategy for building foundational emotional resilience. It should be noted that the gender difference in the effect of PE on ERA may also be related to the types of physical exercise participated in by males and females in the sample (e.g., males tend to participate in competitive sports, while females tend to participate in low-intensity sports) and the unmeasured variables such as parental educational level, which need to be verified in future research.

The finding that ERA consistently mediated the PE-SA link, with a larger effect size than PR, indicates a potential mechanism: physical exercise may reduce social anxiety not primarily by providing social exposure, but by enhancing top-down cognitive control over emotional responses. According to Gross’s process model of emotion regulation, exercise may improve the ability to select and modify situations, deploy attention, and reappraise threatening social cues (Gross, 2002). Neurobiologically, exercise-induced increases in prefrontal cortex activity and hippocampal neuroplasticity may strengthen regulatory capacity (Sun et al., 2023). For left-behind adolescents who experience chronic stress and lack parental co-regulation, this internally focused pathway may be more accessible and effective than peer-mediated pathways.

### 4.2 Reinterpreting the role of peer relationships (PR)

Our results also confirmed that PR significantly mediates the link between PE and SA (H3). Better peer relationships were strongly associated with lower social anxiety, highlighting the mitigating effect of social support, a finding consistent with social support theory (Schwarzer et al., 2004) and prior research on adolescents (Pickering et al., 2020). However, a notable and unexpected finding was that PE did not directly predict PR. This may indicate that the social benefits of exercise are not necessarily automatic. Simply engaging in physical activity, even in a group setting, does not guarantee improved friendships. The non-significant direct path from PE to PR may be attributed to two main factors: first, the quality of social interaction during physical exercise (e.g., most group exercises in the sample are competitive rather than cooperative, which cannot effectively promote peer bonding); second, the pre-existing social anxiety of left-behind adolescents inhibits their active social engagement in group exercise settings, even if they participate in collective physical activities. These factors may lead to the failure of physical exercise to directly improve peer relationships.

When viewed alongside the ERA findings, a clearer picture emerges: PE is associated with an increase in ERA. While the sequential pathway (PE → ERA → PR → SA) was not statistically supported, the data do show that ERA is associated with both higher PR and lower SA in parallel. Thus, ERA may enable better social functioning, but this should not be interpreted as evidence for a sequential causal chain. This aligns with a body of literature demonstrating that individuals with developed ERA exhibit superior social skills and build more positive connections (Zheng and Fang, 2009). Our finding challenges the assumption that physical activity inherently improves peer relations, and instead proposes that emotional regulation could be a prerequisite for exercise-related social benefits (Lin et al., 2024).

The non-significant direct path from PE to PR and the rejection of the sequential mediation (H4) carry important theoretical implications. For left-behind adolescents, the social benefits of physical exercise do not appear automatic but may depend on prior improvements in internal emotional capacities. This pattern aligns with a regulation-first model, where enhanced emotion regulation serves as a prerequisite for translating social opportunities into quality peer relationships. Recent longitudinal evidence on social–emotional competencies supports this interpretation, showing that internal emotional skills underpin subsequent social outcomes (Lin et al., 2024). Thus, our findings refine when exercise-induced social benefits emerge: for vulnerable youth lacking parental co-regulation, building regulatory capacity may be a necessary first step.

### 4.3 What does the rejection of H4 tell us? Decomposing the non-significant chain

The statistical pattern observed for H4 warrants careful theoretical interpretation. On the surface, the non-significant sequential indirect effect (Effect = −0.025, 95% CI [−0.051, 0.027]) appears to contradict the hypothesized cascade. However, the significance of each individual link—PE → ERA (*β* = 0.102, *p* < 0.01), ERA→PR (*β* = 0.498, *p* < 0.001), and PR → SA (*β* = −0.495, *p* < 0.001)—tells a more nuanced story: the sequential logic is empirically supported at each step, but the first step is too small to carry the chain to significance.

This finding carries several implications. First, it suggests that the theoretical model linking exercise to emotion regulation, peer relationships, and social anxiety is not fundamentally flawed. The conceptual chain—that exercise may improve emotional competence, which in turn facilitates better peer connections, which then buffer against social anxiety—remains plausible. Second, it identifies the PE → ERA link as the critical bottleneck in this pathway. For left-behind adolescents, the association between simply participating in physical exercise and developing emotion regulation abilities, while statistically detectable (*β* = 0.102), may be insufficient to initiate a downstream cascade.

Methodologically, this pattern also underscores the importance of statistical power and effect size in mediation analysis. The product of three coefficients is inherently smaller than each individual coefficient; even when all three paths are significant, the overall indirect effect may fail to reach significance if any single path is small. In the present study, the PE → ERA coefficient of 0.102, when multiplied by 0.498 and −0.495, yielded a product (−0.025) whose confidence interval crossed zero.

Theoretically, this suggests that for left-behind adolescents to derive cascading social benefits from exercise, the emotional regulatory gains from exercise may need to be substantially strengthened. This is consistent with a threshold effect interpretation: the association between PE and ERA may need to reach a certain magnitude before it can effectively propagate through peer relationships to reduce social anxiety. Interventions may therefore need to focus not merely on increasing exercise participation, but on deliberately designing exercise programs that explicitly cultivate emotional competencies.

### 4.4 The absence of a sequential chain: a new model

Beyond the identification of the PE → ERA bottleneck, the data also showed that ERA and PR functioned as independent mediators of the PE-SA association. Specifically, the indirect effects via ERA (Effect = −0.034) and via PR (Effect = −0.022) were both significant. This parallel pattern suggests that ERA may have dual beneficial effects: a direct association with lower SA, and an indirect association via improved PR. This observation is consistent with the emotion regulation deficit model of social anxiety (Cisler et al., 2010; Lee and Ju, 2019), which posits that effective emotion regulation directly mitigates anxiety symptoms, independent of social context.

However, it is critical to emphasize that this parallel pattern was not pre-specified; it emerged from post-hoc observation following the rejection of H4. It should therefore be interpreted as hypothesis-generating rather than confirmatory, and requires independent replication in future research. The primary contribution of this study remains the identification of the PE → ERA link as the critical bottleneck in the sequential pathway—not the establishment of a new parallel mediation model.

### 4.5 Implications

#### 4.5.1 Theoretical implications

This study offers several theoretical refinements. First, by decomposing the non-significant chain mediation into its constituent paths, we identify the PE → ERA link as the critical bottleneck in the hypothesized sequential pathway. The significance of all constituent paths suggests that the sequential logic is theoretically viable, but the PE → ERA association in this population (*β* = 0.102) may be too weak to initiate a downstream cascade. This refines theoretical expectations: for vulnerable adolescents, the pathway from exercise to social benefits may require stronger or more targeted cultivation of emotion regulation skills before peer-mediated effects can emerge.

Second, the non-significant direct path from PE to PR, combined with the significant indirect path via ERA, suggests that emotional competence may be a prerequisite for exercise-related social benefits. This aligns with a “regulation-first” model, wherein internal regulatory skills must develop before social opportunities can be effectively leveraged—though this interpretation remains provisional.

Finally, the significant gender moderation provides empirical support for theories calling for gender-sensitive models of mental health intervention.

#### 4.5.2 Practical implications

Our findings offer clear guidance for schools, counselors, and policymakers.

First, strengthening the PE → ERA link as a prerequisite for cascading benefits. The finding that the chain mediation failed due to the small PE → ERA association, despite significant downstream paths (ERA→PR → SA), suggests that interventions should prioritize building the emotional regulatory benefits of exercise. Simply encouraging participation may be insufficient; programs should explicitly teach emotion regulation skills within physical activity settings—for example, by integrating mindfulness, breathing exercises, or cooperative games that encourage emotional awareness (Villodres et al., 2025). For left-behind adolescents who lack parental co-regulation, the pathway from exercise to social benefit is not automatic; it requires intentional cultivation of internal emotional competencies.

Second, tailoring interventions to gender-specific needs. Female left-behind adolescents in our sample derived greater emotional benefits from exercise than males, yet reported lower baseline participation. Targeted programs to encourage and sustain their engagement are important, including creating safe, supportive, and non-judgmental environments. Schools should consider offering diverse activity options—including non-competitive or low-intensity activities—that may be particularly conducive to emotion regulation for girls (Wei et al., 2025).

Finally, recognizing emotion regulation as a prerequisite. School-based mental health programs should acknowledge that building emotional skills may be a necessary first step before social skills interventions can be fully effective for anxious adolescents.

### 4.6 Limitations and future directions

Several limitations should be acknowledged. First, the cross-sectional design precludes causal conclusions. Although we proposed a theoretically driven mediation model, alternative directional paths or reciprocal relationships are equally plausible. Future research should employ multi-wave longitudinal designs or ecological momentary assessment (EMA) to establish temporal precedence and capture dynamic, within-person relationships (Bolger and Laurenceau, 2013).

Second, the modest internal consistency of the ERA and PR scales (*α* < 0.70) is a methodological concern. Low reliability can attenuate correlations and bias indirect effect estimates, Future studies should use more robust, multidimensional measures of emotion regulation and peer relationships and consider incorporating multi-informant reports (e.g., peer nominations, teacher ratings) to permit more precise tests of the proposed parallel mediation model.

And, the pattern observed for H4—non-significant chain mediation despite significant constituent paths—raises an important methodological consideration. The product of three coefficients is inherently smaller than each individual coefficient; thus, detecting a significant sequential indirect effect requires relatively large path coefficients, particularly for the initial predictor-mediator link. The PE → ERA coefficient of 0.102 may have been too small to yield a significant product term, even though the sequential logic was supported at each step. Future studies with larger samples or more sensitive measures of PE and ERA may be better powered to detect such chain effects. Researchers should be mindful that non-significant chain mediation does not necessarily imply a failure of the sequential model; it may instead reflect insufficient magnitude in one or more constituent paths.

Additionally, the relatively small effect sizes observed in this study suggest that physical exercise represents only one of multiple factors associated with social anxiety, and its practical impact should be interpreted within a broader psychosocial context.

## 5 Conclusion

This exploratory cross-sectional study provides preliminary evidence that PE is correlated with lower SA among left-behind adolescents. A key finding is the non-significant sequential mediation (PE → ERA→PR → SA), despite each constituent path being individually significant. This pattern suggests that the PE → ERA association—while statistically detectable—may be too modest to initiate a downstream cascade through peer relationships to social anxiety. The bottleneck in the hypothesized pathway lies not in the sequential logic (which was supported at each step), but in the magnitude of the initial exercise-emotion regulation link.

These findings, while preliminary, offer a provisional theoretical direction: for vulnerable adolescents, achieving cascading social benefits from exercise may require deliberate strengthening of the emotional regulatory gains from physical activity. If replicated in longitudinal research, these findings would suggest that interventions promoting PE for left-behind adolescents should prioritize the cultivation of emotion regulation skills within exercise settings, with particular attention to female adolescents who appear to derive greater emotional benefits.

## Data Availability

The original contributions presented in the study are included in the article/supplementary material, further inquiries can be directed to the corresponding author.
